# Immunostimulation by OX40 Ligand Transgenic Ewing Sarcoma Cells

**DOI:** 10.3389/fonc.2015.00242

**Published:** 2015-10-27

**Authors:** Dajana Reuter, Martin S. Staege, Caspar D. Kühnöl, Jürgen Föll

**Affiliations:** ^1^University Clinic and Polyclinic for Child and Adolescent Medicine, Martin Luther University Halle-Wittenberg, Halle, Germany; ^2^Department of Pediatric Hematology, Oncology and Stem Cell Transplantation, University Hospital Regensburg, Regensburg, Germany

**Keywords:** Ewing sarcoma, immunotherapy, co-stimulation, OX40/OX40L system, tumor necrosis factor (receptor) superfamily

## Abstract

Interleukin-2 (IL-2) transgenic Ewing sarcoma cells can induce tumor specific T and NK cell responses and reduce tumor growth *in vivo* and *in vitro*. Nevertheless, the efficiency of this stimulation is not high enough to inhibit tumor growth completely. In addition to recognition of the cognate antigen, optimal T-cell stimulation requires signals from so-called co-stimulatory molecules. Several members of the tumor necrosis factor superfamily have been identified as co-stimulatory molecules that can augment antitumor immune responses. OX40 (CD134) and OX40 ligand (OX40L = CD252; also known as tumor necrosis factor ligand family member 4) is one example of such receptor/ligand pair with co-stimulatory function. In the present investigation, we generated OX40L transgenic Ewing sarcoma cells and tested their immunostimulatory activity *in vitro*. OX40L transgenic Ewing sarcoma cells showed preserved expression of Ewing sarcoma-associated (anti)gens including lipase member I, cyclin D1 (CCND1), cytochrome P450 family member 26B1 (CYP26B1), and the Ewing sarcoma breakpoint region 1-friend leukemia virus integration 1 (EWSR1-FLI1) oncogene. OX40L-expressing tumor cells showed a trend for enhanced immune stimulation against Ewing sarcoma cells in combination with IL-2 and stimulation of CD137. Our data suggest that inclusion of the OX40/OX40L pathway of co-stimulation might improve immunotherapy strategies for the treatment of Ewing sarcoma.

## Introduction

Ewing sarcomas (or “Ewing family tumors,” EFT) represent bone and/or soft-tissue tumors of uncertain histogenetic origin. The majority of the cases are observed in children and young adults. Today, more than half of the patients with localized EFT can be cured. However, the prognosis for patients with disseminated disease or early relapses remains poor with conventional therapy (1). EFTs are characterized by the expression of tumor-specific oncofusion proteins (2). These fusion proteins are highly tumor specific and might be interesting targets for immunological treatment strategies. However, peptides derived from these proteins have only low binding affinity to common human leukocyte antigen (HLA) class I molecules (3). Using high-density DNA microarrays, we identified additional potential tumor antigens expressed in EFT (4–7). The presence of such tumor-specific antigens alone is not sufficient for the induction of efficient immune responses. Additional co-stimulatory signals are required. We demonstrated that interleukin-2 (IL-2) transgenic EFT cells can induce immune responses against wild-type tumor cells *in vitro* and in a xenotransplantation model (8–10). However, tumor growth was not completely inhibited in this model, suggesting that additional co-stimulatory signals might be required. One group of such co-stimulatory molecules is represented by members of the tumor necrosis factor (TNF) superfamilies (SF) consisting of the superfamily of TNF receptors (TNFRSF) and the corresponding ligands (TNFSF). The eponymic member of the TNFSF has been initially characterized as a factor with antitumor activity in mice (11). TNF is the prototype of a large gene family, which has several immune-regulatory functions and can augment antitumor immune responses (12, 13). Members of the tumor necrosis factor receptor superfamily comprise a group of type I membrane glycoproteins consisting of more than 50 members that have been identified as co-stimulatory molecules that augment antitumor immune responses. Activation of these surface receptors by the natural ligands or by agonistic antibodies leads to different cellular responses ranging from cell differentiation, proliferation, apoptosis, and survival to enhanced production of cytokines and chemokines (13–16). The differential and unique expression of the TNFRSF molecules on cells of the immune system has made these molecules as ideal targets for new immune therapy strategies (13, 15). OX40 (CD134) and CD137 (4-1BB) and their ligands OX40L (CD252) and 4-1BBL are examples of such co-stimulatory molecules. CD137 (4-1BB) is an activation-inducible TNFRSF member expressed on activated T cells (CD8-positive and CD4-positive T cells) and is also expressed on a variety of immune cell lineages including activated natural killer cells, human macrophages, eosinophils, and dendritic cells (17). The natural ligand for CD137 (4-1BBL) is mostly expressed on professional antigen-presenting cells or in inflamed non-hematopoietic tissues (15).

Recently, we analyzed the effects of the CD137/4-1BBL system in our Ewing sarcoma immune-therapy model (10). 4-1BBL transgenic cells or agonistic antibodies against CD137 can induce rejection of varying tumors *in vivo* (18, 19). In our Ewing sarcoma model, we observed modulation of immunosuppressive indoleamine 2,3-dioxygenase 1 (IDO) expression by stimulation of the CD137/4-1BBL system (10). However, engagement of this co-stimulatory system had only limited efficacy for enhancing the immunostimulatory activity of EFT cells (10). The OX40/OX40L system represents another highly interesting co-stimulatory system. OX40 (CD134) was identified as cell surface molecule on activated T cells (20). OX40 is preferentially expressed on CD4-positive T cells (21–23). Optimal antigenic stimulation induces OX40 expression also on CD8-positive T cells (24). The human OX40 molecule has a molecular weight of 50 kDa and is encoded on chromosome 1p36. Murine and human OX40 have only approximately 62% sequence homology in the intracellular domain and <64% in the extracellular domain (25, 26). OX40 is absent from unstimulated peripheral blood mononuclear cells (PBMCs) and most antigen-presenting cells (27). OX40 expression peaks 48 h after stimulation of naive T cells, whereas memory T cells express high levels 4 h after restimulation (28). In contrast to the OX40 receptor, the ligand OX40L (CD252, TNFSF4) is expressed on several professional antigen-presenting cell types, endothelial cells, and activated T cells (29–32). Human OX40L has a molecular weight of 34 kDa and is located on chromosome 1q25 (25, 26). Activation of the OX40 receptor by OX40L or an agonistic antibody leads to increased expression of antiapoptotic molecules and reduced expression of the inhibitory cytotoxic T-lymphocyte antigen 4 (CTLA4) (25, 33, 34). An important aspect of OX40 for antitumor immune responses is the observation that the OX40/OX40L system favors the development of tumor-specific memory T cells and T cells expressing OX40 have been found in tumor-draining lymph node cells and in tumor-infiltrating lymphocytes from patients with various tumors (15, 35). In addition, direct enhancement of cytotoxic T cells by OX40 stimulation has been proposed (36). Therefore, in the present investigation, we established OX40L overexpressing Ewing sarcoma cells for analyzing the effects of OX40 stimulation in our immunotherapy model.

## Materials and Methods

### Gene Expression Analysis and Cloning of OX40L

RNA from cell lines was isolated using TRIzol reagent (Invitrogen, Karlsruhe, Germany) following manufacturer’s protocol. Two micrograms of the RNA was transcribed into cDNA and used as template for polymerase chain reaction (PCR). Reverse transcription of RNA was performed by using the following conditions: 4 μL 5× buffer, 1 μL Oligo-dT12-18 primer, 1 μL dNTP mix (10 mM), 1 μL Revert Aid H-M-MuLV reverse transcriptase (Fermentas, St. Leon Rot, Germany); 37°C, 60 min; and 90°C, 5 min. After reverse transcription, 2 μL cDNA was mixed with 2.5 μL 10× buffer, 1.5 μL MgCl_2_ (25 mM), 0.2 μL Taq-polymerase (Promega, Mannheim, Germany), 0.5 μL dNTP mix (10 mM; Fermentas), 0.25 μL of sequence specific primers (MWG-Biotech AG, Ebersberg, Germany), and 17.8 μL water. The following primer combinations were used: actin beta (ACTB): 5′-GGC ATC GTG ATG GAC TCC G-3′ and 5′-GCT GGA AGG TGG ACA GCG A-3′; cyclin D1 (CCND1): 5′-AAC TAC CTG GAC CGC TTC CT-3′ and 5′-CCA CTT GAG CTT GTT CAC CA-3′; CD99: 5′-TCC TCC GGT AGC TTT TCA GA-3′ and 5′-TCC CCT TGT TCT GCA TTT TC-3′; OX40L (primer combination 1): 5′-aac tcg agT ATC GCA CGT TCC CCT T-3′ (nucleotides in lower case: *Xho*I restriction site) and 5′-aac cgc ggC CAG GAT CTG CTT-3′ (nucleotides in lower case: *Sac*II restriction site); OX40L (primer combination 2): 5′-GTG AAT GGC GGA GAA CTG AT-3′ and 5′-GCC AGG ATC TGC TTC TTG TC-3′; cytochrome P450 26B1 (CYP26B1): 5′-TGA CAG GAT CCC TGT GTT GT-3′ and 5′-CCA ACA TCG AAA GTG CTT CA-3′; enhanced green fluorescent protein (eGFP): 5′-ACG TAA ACG GCC ACA AGT TC-3′ and 5′-AAG TCG TGC TGC TTC ATG TG-3′; Janus kinase 1 (JAK1): 5′-TGT AAG GAG CTG GCT GAC CT-3′ and 5′-CAC CTG CTC CCC TGT ATT GT-3′; lipase H (LIPH): 5′-GAT GGC TGG GGA GAA TTA CA-3′ and 5′-TGG ATT CTG TGG TGT TTC CA-3′; lipase I (LIPI): 5′-TCC GAG AAT AGA GAC CAT TCT GA-3′ and 5′-GCT CTC TGG TGG TTG CAT TT-3′; neomycin resistance cassette (NeoR): 5′-AGA CAA TCG GCT GCT CTG AT-3′ and 5′-AGT GAC AAC GTC GAG CAC AG-3′. The PCR conditions were as follows: 94°C, 30 s; 60°C, 45 s; and 72°C, 45 s (35 cycles). Each PCR program started with a denaturation step (95°C, 5 min) and was finished with 72°C for 5 min followed by cooling down to 4°C. The PCR products were subjected to agarose gel (1.5%) electrophoresis in the presence of ethidium bromide. DNA microarray data were visualized by using Genesis (37). DNA microarray data from EFT cell lines (4, 38), human embryonic kidney 293 (HEK293) cells (39), neuroblastoma (NB) cell lines (4), acute lymphatic leukemia (ALL) cell lines (40, 41), acute myeloid leukemia (AML) cell lines (42), Hodgkin’s lymphoma (HL) cell lines (43), Epstein–Barr virus-immortalized lymphoblastoid cell lines (LCL) (44), and normal PBMCs (45) were used. For cloning of OX40L, cDNA was amplified by using the OX40L primer combination 1. PCR products and vector pIRES2-eGFP (Clontech, Mountain View, CA, USA) were digested with *Xho*I and *Sac*II. After agarose gel purification, ligation, and transformation into *Escherichia coli* XL1-Blue, individual clones were sequenced by using primers 5′-CAA GTC TCC ACC CCA TTG AC-3′, 5′-GTG AAG ATG GAA AGG GTC CA-3′, 5′-aac cgc ggC CAG GAT CTG CTT-3′, and 5′-CAG GGC ATG GAT TCT TCA TT-3′. For sequencing, a 10 μL sequencing mix was used that contained 0.5 μL gene-specific sequencing primers (10 μM), 4.0 μL BigDyeTerminator Cycle Sequencing Kit mix (Applied Biosystems, Foster City, CA, USA), and 10–30 ng DNA. Sequence analysis was performed using ABI Prism™ 310 Genetic Analyzer (Applied Biosystems). A clone with complete error-free OX40L open reading frame was used for further analysis. This clone differs from the reference sequence by a T to C transition in the 3′-untranslated region (corresponding to residue 738 in reference sequence NM_003326).

### Cells and Cell Culture

A673 cells and HEK293 cells were obtained from the American Type Culture Collection (Manassas, VA, USA). SK-N-MC cells and SH-SY5Y cells were obtained from the Deutsche Sammlung für Mikroorganismen und Zellkulturen (Brunswick, Germany). 4-1BBL transgenic A673 cells and stimulation of PBMCs with anti-CD137 antibodies (clone 26G6; a kind gift from R. Mittler) was described elsewhere (10). PBMCs were isolated from healthy donors with informed consent and approval by the local ethics committee as described (45). Cells were cultured in RPMI-1640 medium supplemented with 10% fetal calf serum and penicillin and streptomycin. For selection of transgenic cells, medium was supplemented with 400 μg/mL geneticin sulfate. PBMCs from HLA-A1,A2 positive donors were isolated as described (44, 45). Stimulation of PBMCs and enzyme-linked immunospot (ELISPOT) analysis was performed as described (7) by using an interferon gamma ELISPOT kit (Becton-Dickinson, Heidelberg, Germany). Transfection of cells was performed using jet PEI (Qbiogene, Carlsbad, CA, USA). MACS separation was performed using anti-PE microbeads (Miltenyi, Bergisch Gladbach, Germany). Statistical analysis was performed with Microsoft Excel 2010 (Microsoft, Redmond, WA, USA).

### Flow Cytometry

Phycoerythrin (PE)-labeled mouse anti-human-OX40L antibodies were purchased from Anzell (Bayport, USA). Mouse IgG1 isotype control was purchased from Becton-Dickinson. Flow cytometry was performed as described (40), and cells were analyzed on a FACScan flow cytometer (Becton-Dickinson) equipped with CellQuest Pro software (Becton-Dickinson).

## Results

### Cloning of OX40L into a Mammalian Expression Vector

**Figure 1:**
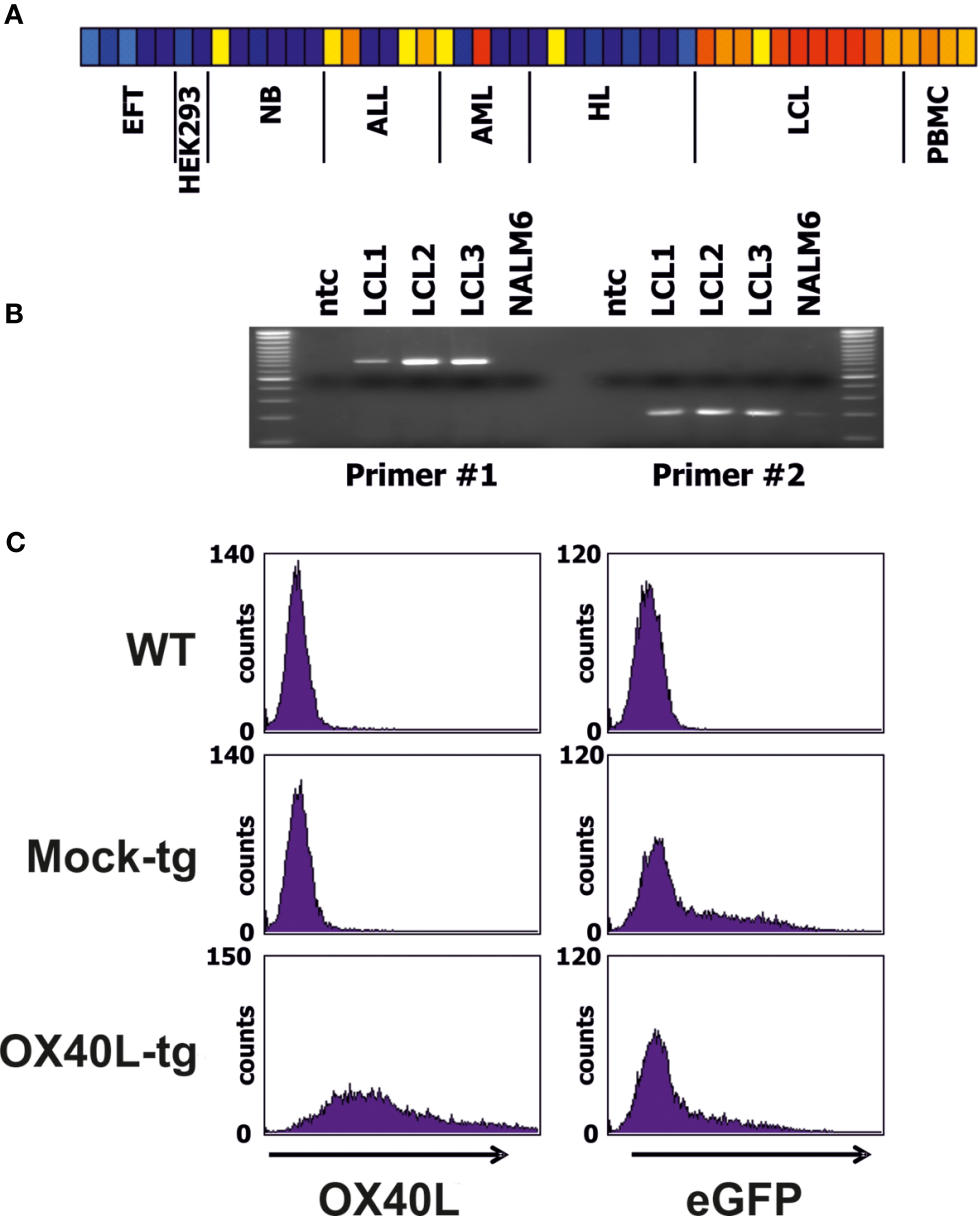
**Expression pattern and cloning of OX40L**. **(A)** DNA microarray data from a panel of cell lines and normal PBMCs were analyzed for expression of OX40L. Presented is a heat map of OX40L signal intensities form EFT cell lines, HEK293 cells, neuroblastoma (NB) cell lines, acute lymphoid (ALL) and myeloid (AML) leukemia cell lines, Hodgkin’s lymphoma (HL) cell lines, lymphoblastoid cell lines (LCL), and normal PBMCs. Red and yellow correspond to high signal intensities and blue corresponds to low signal intensities. From left to right, the following cell lines are shown: A673 (two samples), SK-N-MC, SBSR-AKS (38) (two samples), HEK293 (two samples), CHP-126 (two samples), SiMa, SH-SY5Y (three samples), RPMI, Loucy, Karpas, CALL2, 697, NALM6, U937, Kasumi, KG1, HL60, SKNO, L-428, HD-MyZ, KM-H2, HDLM-2, L-1236 (three samples), L-540 (two samples), 11 independent LCL, and four independent PBMC samples. **(B)** Expression of OX40L was analyzed by RT-PCR in three LCL and NALM6 ALL cells. Two different primer combinations were used. ntc = no template control. **(C)** After amplification of OX40L with primer combination 1 (see Materials and Methods), PCR products from LCL were cloned into vector pIRES2-eGFP. Functionality of the vector was assessed by transfection of HEK293 cell. Empty pIRES2-eGFP without OX40L (Mock) served as control. Transfected cells were stained with anti-OX40L-PE antibodies and analyzed by flow cytometry. eGFP served as marker for transfected cells.

We screened DNA microarray data of varying cell types for samples with high expression of OX40L. Highest expression was observed in lymphoblastoid B cell lines (LCL) (Figure 1A). High expression of OX40L in LCL was validated by RT-PCR (Figure 1B). All analyzed LCL expressed OX40L. In contrast, no expression of OX40L was detectable in the B-cell leukemia cell line NALM6 (Figure 1B). Primer combination 1 amplifies the complete open reading frame of OX40L. The amplificate from LCL with this primer combination was eluted from agarose gels and cloned into vector pIRES2-eGFP. Functionality of the vector was assessed by transient transfection of HEK293 cells (Figure 1C). Wild-type HEK293 cells are negative for OX40L (Figure 1A). After transfection with the OX40L-containing vector, HEK293 cells clearly expressed OX40L on the surface (Figure 1C). HEK293 cells that had been transfected with the empty vector were not stained with antibodies against OX40L. Green fluorescence of eGFP indicated that these cells were successfully transfected with similar efficiency as the OX40L transfected cells (Figure 1C).

### Generation of OX40L Transgenic Ewing Sarcoma Cells

**Figure 2:**
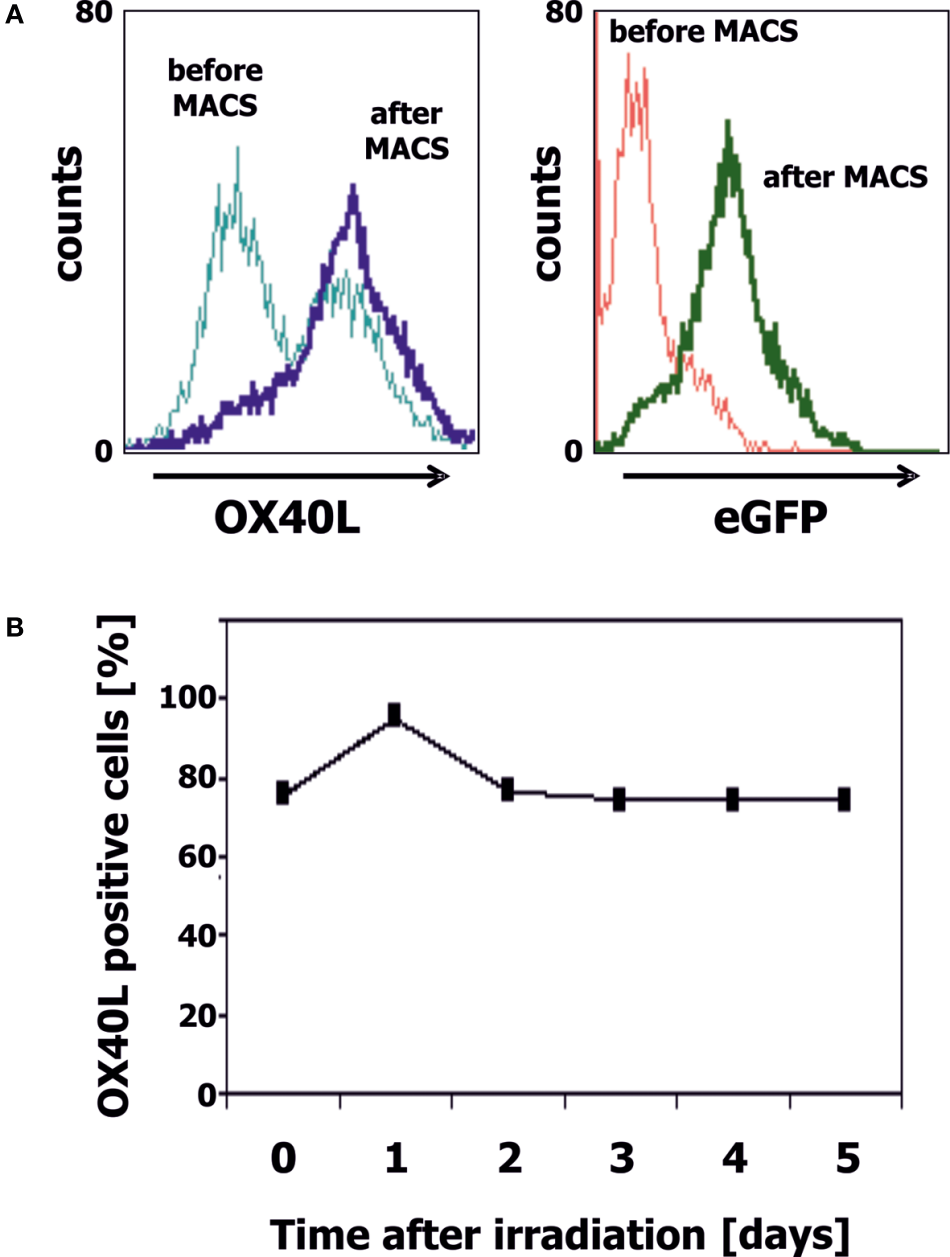
**Generation of OX40L transgenic EFT cells**. **(A)** A673 EFT cells were transfected with OX40L in vector pIRES2-eGFP. Transfected cells were stained with anti-OX40L-PE antibodies and analyzed by flow cytometry. eGFP served as marker for transfected cells. Transgenic cells were enriched by using anti-PE-microbeads. After enrichment, nearly all cells stained positive for OX40L (dark blue line) and eGFP (green line). **(B)** A673 EFT cells were irradiated with 30 Gy and cultured for 5 days. Stability of OX40L expression after irradiation was assessed by staining with anti-OX40L-PE antibodies and flow cytometry.

**Figure 3:**
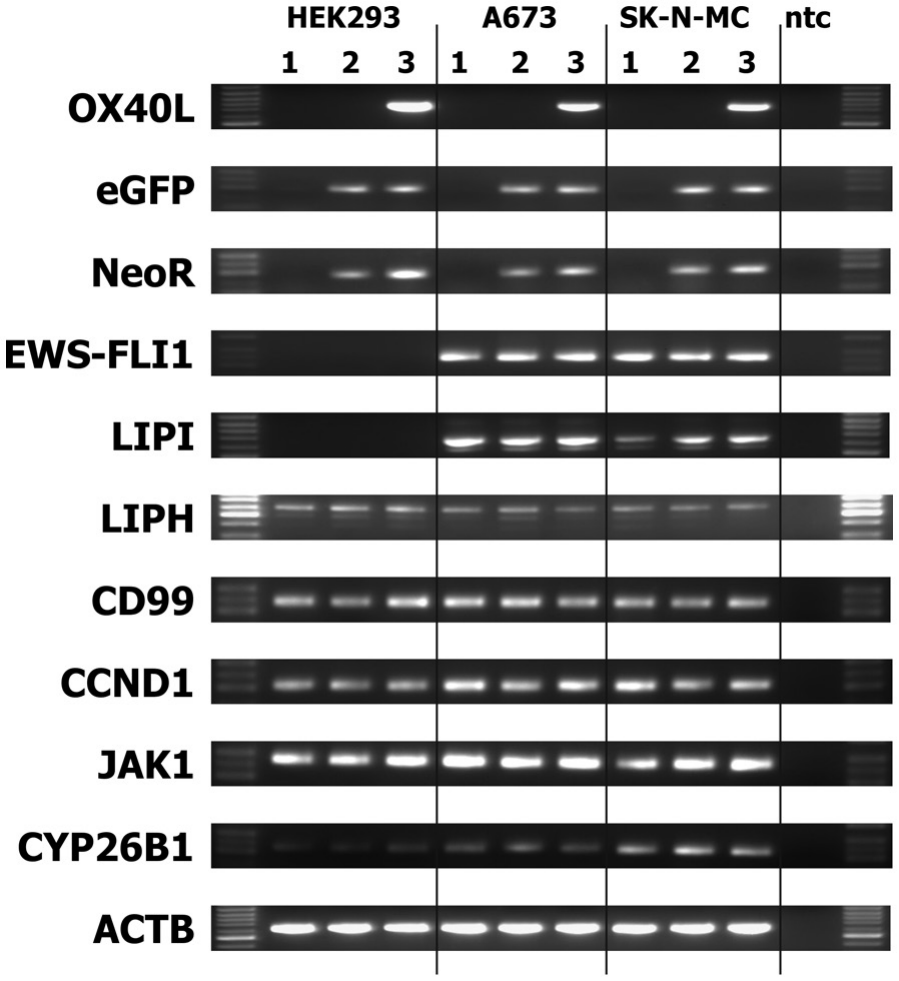
**Stability of EFT makers in OX40L transgenic EFT cells**. Expression of the indicated markers was assessed by RT-PCR in cells without transfection (1), after transfection with empty pIRES-eGFP vector (2), or after transfection with OX40L in vector pIRES-eGFP (3). Actin beta (ACTB) served as housekeeping control. The neomycin-resistance cassette (NeoR) and enhanced green fluorescent protein (eGFP) served as markers for the presence of the vector in the cells. Transfected and wild-type Ewing sarcoma cells expressed the Ewing sarcoma-specific EWS-FLI1 oncofusion transcripts as well as the Ewing sarcoma-associated factors lipase I (LIPI), lipase H (LIPH), CD99, cyclin D1 (CCND1), janus kinase 1 (JAK1), and cytochrome P450 family member 26B1 (CYP26B1).

We transfected cells from the Ewing sarcoma cell lines A673 and SK-N-MC with OX40L in vector pIRES2-eGFP. Transfected cells were selected with geneticin sulfate and further enriched by staining with PE-labeled anti-OX40 antibodies and immunomagnetic beads directed at PE. After magnet-activated cell sorting (MACS), more than 80% of the cells expressed eGFP and OX40L (Figure 2A). For immunotherapy with transgenic tumor cells, it is necessary that the tumor cells can be irradiated without functional impairments. Therefore, we tested the stability of OX40L after irradiation. As shown in Figure 2B, OX40L remained stable after irradiation for at least 5 days. Another prerequisite of transgenic tumor cells for immunotherapy is the stability of tumor antigen expression in these cells. We tested the presence of Ewing sarcoma-associated transcripts in the transgenic cells. OX40L transgenic Ewing sarcoma cells showed the same gene expression pattern as wild-type cells or mock-transfected cells (Figure 3). In contrast to wild-type cells, OX40L transgenic cells and mock transgenic cells expressed transcripts for the vector marker eGFP and the NeoR. Importantly, transgenic Ewing sarcoma cells stably expressed the Ewing sarcoma-specific oncofusion protein EWS-FLI1 (Figure 3). Other genes typically expressed in Ewing sarcoma cells that remained stably expressed after transfection included the putative cancer/testis antigen LIPI (4, 5), LIPH (46), the surface glycoprotein CD99 (47), cyclin D1 [CCND1 (4, 48)], janus kinase 1 [JAK1 (4)], and the retinoic acid metabolizing cytochrome P450 member 26B1 [CYP26B1 (4, 49)] (Figure 3).

### Assessment of the Immunostimulatory Activity of OX40L Transgenic Ewing Sarcoma Cells

**Figure 4:**
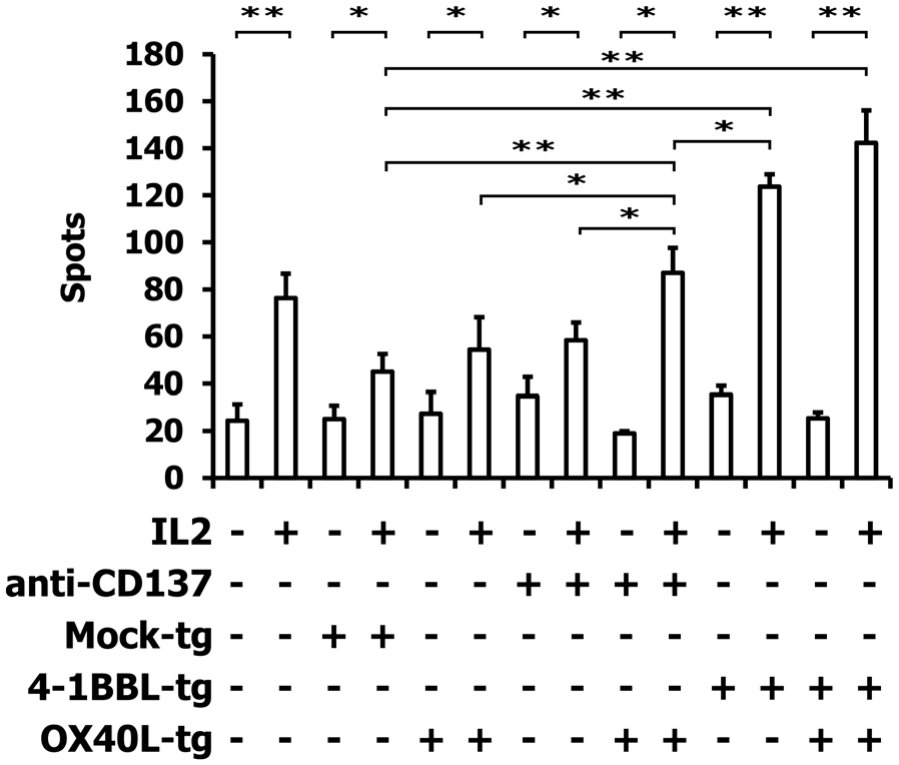
**ELISPOT analysis of PBMCs after stimulation with A673 EFT cells**. HLA-matched PBMCs were incubated together with A673 EFT cells in combination with the indicated immune stimuli. After 6 days, reactivity against A673 wild-type cells was assessed by interferon gamma ELISPOT analysis. The highest numbers of spots were obtained after stimulation with the combination of OX40L transgenic and 4-1BBL transgenic cells. Presented are means and standard deviations from triplicates from a representative experiment (*N* = 3). Asterisks indicate statistical significance (*p < 0.05; **p < 0.01; Student’s *t*-test).

**Figure 5:**
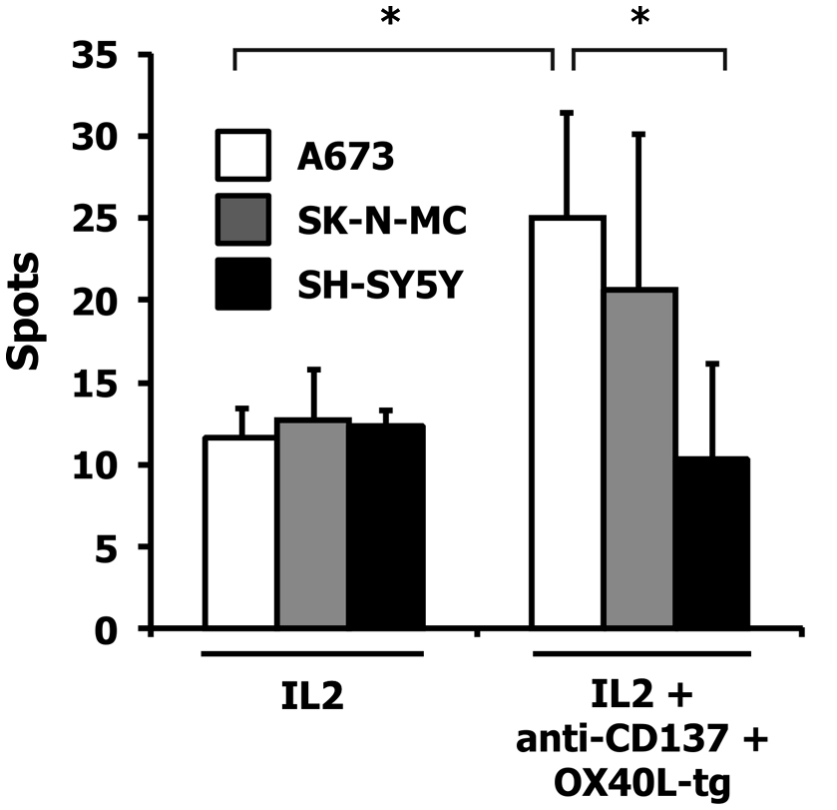
**Specificity of the induced immune response**. HLA-matched PBMCs were incubated together with A673 EFT cells in combination with the indicated immune stimuli. After 6 days, reactivity against the indicated wild-type cell lines was assessed by interferon gamma ELISPOT analysis. The highest numbers of spots were obtained after restimulation of A673/anti-CD137/OX40L-primed cells with A673 cells. Presented are means and standard deviations from triplicates from a representative experiment (*N* = 3). Asterisks indicate statistical significance (*p* < 0.05; Student’s *t*-test).

For the following experiments, we used A673 Ewing sarcoma cells with the partial HLA type A1,A2 for which HLA-matched PBMCs are easily available (9, 10). HLA-matched (HLA-A1,A2 positive) PBMCs were stimulated with A673 cells in the presence or absence of OX40L transgenic cells, interleukin 2, and antibodies against CD137 or 4-1BBL transgenic A673 cells. Reactivity of the primed cells against wild-type A673 cells was assessed by ELISPOT analysis (Figure 4). As expected (9, 10), the presence of interleukin 2 increased the number of cells that reacted with the sarcoma cells. In combination with interleukin 2, OX40L-transfected A673 cells marginally increased the reactivity of the primed cells compared to mock-transfected cells. Addition of antibodies against CD137 enhanced this effect significantly. In the presence of interleukin 2, the combination of anti-CD137 stimulation and OX40L transfected cells showed significantly enhanced immune stimulation in comparison to anti-CD137 antibodies or OX40L-transfected cells alone. The combination of OX40L transgenic cells with 4-1BBL transgenic cells showed a trend for higher stimulatory activity compared to 4-1BBL transgenic cells alone (Figure 4). After priming in the presence of OX40L transgenic A673 cells, the cells showed a higher specificity for A673 cells than for SK-N-MC cells or the neuroblastoma cell line SH-SY5Y (Figure 5).

## Discussion

The role of cancer immunotherapy that boosts the extraordinary power of our immune system to detect and destroy cancer cells still remains unclear. Acquired adaptive cancer immunotherapy regimens represent the most promising new treatment strategies, which have the ability to detect and kill cancer cells specifically and which have the potential to achieve a long-lasting antitumor response. Although tumor cells express tumor-specific antigens that can be recognized and targeted by T cells, the tumor produces different molecular and cellular mechanisms that reduce the ability of the immune system to recognize or kill tumor cells (50). There are many different mechanisms in the tumor microenvironment that suppress ongoing T-cell functions and enable tumor escape (51, 52). For instance, the tumor microenvironment can reduce activation of T cells, tumor cells can escape immune recognition by downregulation of tumor-associated antigens or antigen-presenting HLA molecules, tumor cells can produce antigen-loss variants, tumor cells can secrete immunosuppressive factors (e.g., indoleamine-2,3-dioxygenase), and co-stimulatory signals can be absent from antigen-presenting cells (53–57). Naive T cells require a strong interaction between the T-cell receptor and antigen-presenting HLA molecules (signal 1) and binding of co-stimulatory molecules expressed on the surface of antigen-presenting cells (signal 2) for optimal activation (58). In the absence of a co-stimulatory signal, T cells typically enter a state of anergy or paralysis (59). Some members of the TNFRSF have been identified as co-stimulatory molecules that augment antitumor immune responses. Activation of these surface receptors by their natural ligands or by agonistic antibodies leads to different cellular responses ranged from cell differentiation, proliferation, apoptosis, and survival to enhanced production of cytokines and chemokines (11, 13, 15, 16). The expression of the TNFRSF molecules on cells of the immune system has made these molecules as ideal targets for new immune therapy strategies (14, 15). OX40 (CD134) and CD137 (4-1BB) and their ligands OX40L (CD252) and 4-1BBL are examples of such co-stimulatory effective molecules.

We have shown in previous studies that transgenic expression of IL-2 on EFT cells enhances the immunostimulatory activity but could not completely inhibit the growth of the tumor cells (8, 9). Addition of transgenically expressed co-stimulatory molecules on the surface of tumor cells or stimulation with agonistic antibody against the co-stimulatory receptor may enhance the cytotoxic effect of activated T cells (10, 60). In the present study, we present preliminary data that OX40L transgenic EFT cells not only preserve expression of typical Ewing sarcoma-associated antigens but also might enhance the immune response against EFT cells in combination with IL-2 and stimulation of CD137.

The presence of OX40-positive T cells at sites of tumor metastases suggests that engagement of OX40 by OX40L or agonistic antibodies may enhance function of tumor-reactive T cells. In various studies, different tumor cells transfected with OX40L were used as vaccines to induce tumor-specific antitumor immunity [for review, see Ref. (14, 15, 61)]. Andarini et al. treated subcutaneous tumors of melanoma, Lewis lung carcinoma, and adenocarcinoma with intratumoral injection of tumor cells expressing mouse OX40L (62). It was shown that the treatment of tumor-bearing mice with tumor cells expressing OX40L induced significant suppression of tumor growth and enhanced survival of the treated mice (21). Similar results were found with EL4 lymphoma cells or with C26 colon carcinoma cells in combination with stimulation of APC function with granulocyte–monocyte colony-stimulating factor [GM-CSF (15)]. In all these experiments, both CD4-positive and CD8-positive T cells were required for the induction of antitumor immunity and both CD4-positive and CD8-positive tumor-infiltrating T cells (TILs) expressed OX40. Nevertheless, it was unclear whether OX40-mediated signaling in CD8-positive T cells might have been required to induce their cytotoxic effector function (21). It is possible that activation of the OX40 receptor increases the function of tumor-specific CD4-positive T cell and allows more efficient effector function as well as the generation of CD8-positive T-cell memory (63, 64). Furthermore, in several preclinical models, treatment of tumor-bearing hosts with anti-OX40 agonistic antibodies or OX40L-Fc fusion protein resulted in a significant tumor regression [for review, see Ref. (21)]. In these studies, it was suggested that activation of OX40 receptor by agonistic antibody or OX40L transgenic tumor cells pushes regulatory T cells (Treg) in suppressing or depletion depending on the context of simulation and the cytokine milieu (65). This activation of OX40 on different T cells with agonistic antibody or OX40L-expressing tumor cells may lead to decreased inhibitory effects mediated by Treg cells and thereby might promote antitumor responses of CD8-positive T cell which is necessary to maintain long-term antitumor responses. It is possible that several different mechanisms are important for the antitumor effects mediated through the activation of the OX40 receptor on T cells (61).

It is unlikely that the use of only one co-stimulatory molecule will be sufficient for generating immunotherapy strategies to cure patients with different tumor types. However, the use of combinations of several co-stimulatory molecules may be a more effective strategy for producing immunotherapy against cancer. In this context, Cuadros et al. showed a potential benefit of anti-CD137 and anti-OX40 antibodies in enhancing the immune responses in combination with dendritic cell-based vaccines in a Her-2/neu tumor model. The authors showed that joint co-stimulation with anti-CD137 and anti-OX40 agonistic antibodies induce strong effector immune responses depending on both CD4-positive and CD8-positive T cells (66). The combination of activation of two co-stimulatory molecules induces a strong effector immune response by primary CD8-positive T cells that is sufficient to attack established tumors, induce CD4-positive T-cell responses, and generate tumor-specific T-cell memory (66). Furthermore, Pan et al. successfully treated metastatic colon carcinomas with anti-CD137 agonistic antibodies together with IL-12 transfected tumor cells (67). However, the success was limited to small tumors. The addition of anti-OX40 agonistic antibodies to the immunotherapy protocol improved the success also to greater tumors on established colon carcinomas. This triple co-stimulatory combination therapy induced a high CTL activity in the TILs against parenteral tumors, and this effect was partly cell dependent on CD4-positive T cells. These observations suggested that anti-OX40 antibodies enhanced the helper function of CD4-positive T cells that increased the number or activity of CD8-positive T cells against the tumors (67).

We have shown that OX40L-expressing Ewing sarcoma cells preserved the expression of typical Ewing sarcoma-associated antigens and are practicable for immunotherapy protocols with transgenic tumor cells. The stimulated PBMCs exerted some specificity for the tumor cells that were used for stimulation. It remains unclear which antigens are recognized and which cell types are responsible for the effects. In our previous investigations, we observed activation of T and NK cells by IL-2 transgenic Ewing sarcoma cells (9). The high sensitivity of Ewing sarcoma cells for NK cell-mediated lysis (68, 69) might also be responsible for the higher “specificity” of Ewing sarcoma-activated PBMCs for these stimulatory cells. Whether antigenic peptides in combination with major histocompatibility complex (MHC) molecules are recognized by T cells or whether activated NK cells are triggered by receptors like CD226 or CD314 requires further investigations. Our data suggest that OX40L-expressing tumor cells might enhance immune response against Ewing sarcoma cells in combination with IL-2 and activation of the CD137/4-1BBL co-stimulatory pathway. The inclusion of the OX40/OX40L pathway in co-stimulation immunotherapy protocols might improve the immunotherapy strategies against Ewing sarcoma or the development of tumor vaccines.

## Conflict of Interest Statement

The authors declare that the research was conducted in the absence of any commercial or financial relationships that could be construed as a potential conflict of interest.

## References

[1] Burdach S van Kaick B Laws HJ Ahrens S Haase R Körholz D Allogeneic and autologous stem-cell transplantation in advanced Ewing tumors. An update after long-term follow-up from two centers of the European Intergroup study EICESS. Stem-Cell Transplant Programs at Düsseldorf University Medical Center, Germany and St. Anna Kinderspital, Vienna, Austria. Ann Oncol (2000) 11:1451–62. 11142486 10.1023/a:1026539908115

[2] Staege MS Max D Genetics and epigenetics of the TET-ETS translocation network. Genet Epigenet (2009) 2:1–15.

[3] Pfeifle C Reinhardt K Heins S Burdach S Staege MS. Development and characterization of HAT-sensitive Ewing tumour cells for immunotherapy. Anticancer Res (2009) 29:4489–96. 20032396

[4] Staege MS Hutter C Neumann I Foja S Hattenhorst UE Hansen G DNA microarrays reveal relationship of Ewing family tumors to both endothelial and fetal neural crest-derived cells and define novel targets. Cancer Res (2004) 64:8213–21. 10.1158/0008-5472.CAN-03-4059 15548687

[5] Foell JL Hesse M Volkmer I Schmiedel BJ Neumann I Staege MS. Membrane-associated phospholipase A1 beta (LIPI) is an Ewing tumour-associated cancer/testis antigen. Pediatr Blood Cancer (2008) 51:228–34. 10.1002/pbc.21602 18435455

[6] Max D Hesse M Volkmer I Staege MS. High expression of the evolutionarily conserved alpha/beta hydrolase domain containing 6 (ABHD6) in Ewing tumors. Cancer Sci (2009) 100:2383–9. 10.1111/j.1349-7006.2009.01347.x 19793082 PMC11158961

[7] Mahlendorf DE Staege MS. Characterization of Ewing sarcoma associated cancer/testis antigens. Cancer Biol Ther (2013) 14:254–61. 10.4161/cbt.23298 23291981 PMC3595308

[8] Staege MS Gorelov V Bulankin A Fischer U Dumon K Hohndorf L Stable transgenic expression of IL-2 and HSV1-tk by single and fusion tumor cell lines bearing EWS/FLI-1 chimeric genes. Pediatr Hematol Oncol (2003) 20:119–40. 10.1080/0880010390158612 12554523

[9] Staege MS Hansen G Baersch G Burdach S. Functional and molecular characterization of interleukin-2 transgenic Ewing tumor cells for in vivo immunotherapy. Pediatr Blood Cancer (2004) 43:23–34. 10.1002/pbc.20013 15170886

[10] Max D Kühnöl CD Burdach S Niu L Staege MS Föll JL. Indoleamine-2,3-dioxygenase in an immunotherapy model for Ewing sarcoma. Anticancer Res (2014) 34:6431–41. 25368243

[11] Carswell EA Old LJ Kassel RL Green S Fiore N Williamson B. An endotoxin-induced serum factor that causes necrosis of tumors. Proc Natl Acad Sci U S A (1975) 72:3666–70. 10.1073/pnas.72.9.3666 1103152 PMC433057

[12] Wiemann B Starnes CO. Coley’s toxins, tumor necrosis factor and cancer research: a historical perspective. Pharmacol Ther (1994) 64:529–64. 10.1016/0163-7258(94)90023-X 7724661

[13] Croft M The TNF family in T cell differentiation and function – unanswered questions and future directions. Semin Immunol (2014) 26:183–90. 10.1016/j.smim.2014.02.005 24613728 PMC4099277

[14] Moran AE Kovacsovics-Bankowski M Weinberg AD. The TNFRs OX40, 4-1BB, and CD40 as targets for cancer immunotherapy. Curr Opin Immunol (2013) 25:230–7. 10.1016/j.coi.2013.01.004 23414607 PMC3815601

[15] Foell J Hewes B Mittler RS. T cell costimulatory and inhibitory receptors as therapeutic targets for inducing anti-tumor immunity. Curr Cancer Drug Targets (2007) 7:55–70. 10.2174/156800907780006841 17305478

[16] Mittler RS Foell J McCausland M Strahotin S Niu L Bapat A Anti-CD137antibodies in the treatment of autoimmune disease and cancer. Immunol Res (2004) 29:197–208. 10.1385/IR:29:1-3:197 15181282

[17] Watts TH. TNF/TNFR family members in costimulation of T cell responses. Annu Rev Immunol (2005) 23:23–68. 10.1146/annurev.immunol.23.021704.115839 15771565

[18] Melero I Shuford WW Newby SA Aruffo A Ledbetter JA Hellstrom KE Monoclonal antibodies against the 4-1BB T-cell activation molecule eradicate established tumors. Nat Med (1997) 3:682–5. 10.1038/nm0697-682 9176498

[19] Wilcox RA Flies DB Zhu G Johnson AJ Tamada K Chapoval AI Provision of antigen and CD137 signaling breaks immunological ignorance, promoting regression of poorly immunogenic tumors. J Clin Invest (2002) 109:651–9. 10.1172/JCI0214184 11877473 PMC150893

[20] Paterson DJ Jefferies WA Green JR Brandon MR Corthesy P Puklavec M Antigens of activated rat T lymphocytes including a molecule of 50,000 Mr detected only on CD4 positive T blasts. Mol Immunol (1987) 24:1281–90. 10.1016/0161-5890(87)90122-2 2828930

[21] Sugamura K Ishii N Weinberg AD Therapeutic targeting of the effector T-cell co-stimulatory molecule OX40. Nat Rev Immunol (2004) 4:420–31. 10.1038/nri1371 15173831

[22] Taraban VY Rowley TF O’Brien L Chan HT Haswell LE Green MH Expression and costimulatory effects of the TNF receptor superfamily members CD134 (OX40) and CD137 (4-1BB), and their role in the generation of anti-tumor immune responses. Eur J Immunol (2002) 32:3617–27. 10.1002/1521-4141(200212)32:12<3617::AID-IMMU3617>3.0.CO;2-M 12516549

[23] Weinberg AD Vella AT Croft M. OX-40: life beyond the effector T cell stage. Semin Immunol (1998) 10:471–80. 10.1006/smim.1998.0146 9826580

[24] Bansal-Pakala P Halteman BS Cheng MH Croft M. Costimulation of CD8 T cell responses by OX40. J Immunol (2004) 172:4821–5. 10.4049/jimmunol.172.8.4821 15067059

[25] Croft M. Co-stimulatory members of the TNFR family: keys to effective T-cell immunity? Nat Rev Immunol (2003) 3:609–20. 10.1038/nri1148 12974476

[26] Gruss HJ Dower SK. Tumor necrosis factor ligand superfamily: involvement in the pathology of malignant lymphomas. Blood (1995) 85:3378–404. 7780126

[27] Godfrey WR Fagnoni FF Harara MA Buck D Engleman EG. Identification of a human OX-40 ligand, a costimulator of CD4+ T cells with homology to tumor necrosis factor. J Exp Med (1994) 180:757–62. 10.1084/jem.180.2.757 7913952 PMC2191595

[28] Gramaglia I Weinberg AD Lemon M Croft M. Ox-40 ligand: a potent costimulatory molecule for sustaining primary CD4 T cell responses. J Immunol (1998) 161:6510–7. 9862675

[29] Ohshima Y Tanaka Y Tozawa H Takahashi Y Maliszewski C Delespesse G. Expression and function of OX40 ligand on human dendritic cells. J Immunol (1997) 159:3838–48. 9378971

[30] Miura S Ohtani K Numata N Niki M Ohbo K Ina Y Molecular cloning and characterization of a novel glycoprotein, gp34, that is specifically induced by the human T-cell leukemia virus type I transactivator p40 tax. Mol Cell Biol (1991) 11:1313–25. 10.1128/MCB.11.3.1313 1996093 PMC369402

[31] Murata K Ishii N Takano H Miura S Ndhlovu LC Nose M Impairment of antigen-presenting cell function in mice lacking expression of OX40 ligand. J Exp Med (2000) 191:365–74. 10.1084/jem.191.2.365 10637280 PMC2195745

[32] Imura A Hori T Imada K Ishikawa T Tanaka Y Maeda M The human OX40/gp34 system directly mediates adhesion of activated T cells to vascular endothelial cells. J Exp Med (1996) 183:2185–95. 10.1084/jem.183.5.2185 8642328 PMC2192546

[33] Rogers PR Song J Gramaglia I Killeen N Croft M. OX40 promotes Bcl-xL and Bcl-2 expression and is essential for long-term survival of CD4 T cells. Immunity (2001) 15:445–55. 10.1016/S1074-7613(01)00191-1 11567634

[34] Prell RA Evans DE Thalhofer C Shi T Funatake C Weinberg AD. OX40-mediated memory T cell generation is TNF receptor-associated factor 2 dependent. J Immunol (2003) 171:5997–6005. 10.4049/jimmunol.171.11.5997 14634111

[35] Weinberg AD Rivera MM Prell R Morris A Ramstad T Vetto JT Engagement of the OX-40 receptor in vivo enhances antitumor immunity. J Immunol (2000) 164:2160–9. 10.4049/jimmunol.164.4.2160 10657670

[36] Kjaergaard J Tanaka J Kim JA Rothchild K Weinberg A Shu S. Therapeutic efficacy of OX-40 receptor antibody depends on tumor immunogenicity and anatomic site of tumor growth. Cancer Res (2000) 60:5514–21. 11034096

[37] Sturn A Quackenbush J Trajanoski Z. Genesis: cluster analysis of microarray data. Bioinformatics (2002) 18:207–8. 10.1093/bioinformatics/18.1.207 11836235

[38] Richter GH Plehm S Fasan A Rössler S Unland R Bennani-Baiti IM EZH2 is a mediator of EWS/FLI1 driven tumor growth and metastasis blocking endothelial and neuro-ectodermal differentiation. Proc Natl Acad Sci U S A (2009) 106:5324–9. 10.1073/pnas.0810759106 19289832 PMC2656557

[39] Hutter C Burdach S Staege MS Characterization of EWS/Fli-1 induced gene expression profiles in HEK293 cells. Klin Padiatr (2003) 215:193. 10.1055/s-2003-39371 12838936

[40] Wernicke CM Richter GH Beinvogl BC Plehm S Schlitter AM Bandapalli OR MondoA is highly overexpressed in acute lymphoblastic leukemia cells and modulates their metabolism, differentiation and survival. Leuk Res (2012) 36:1185–92. 10.1016/j.leukres.2012.05.009 22748921

[41] Metzler M Staege MS Harder L Mendelova D Zuna J Fronkova E Inv(11)(q21q23) fuses MLL to the Notch co-activator mastermind-like 2 in secondary T-cell acute lymphoblastic leukemia. Leukemia (2008) 22:1807–11. 10.1038/leu.2008.50 18337764

[42] Berg T Fliegauf M Burger J Staege MS Liu S Martinez N Transcriptional upregulation of p21/WAF/Cip1 in myeloid leukemic blasts expressing AML1-ETO. Haematologica (2008) 93:1728–33. 10.3324/haematol.13044 18790797

[43] Staege MS Banning-Eichenseer U Weissflog G Volkmer I Burdach S Richter G Gene expression profiles of Hodgkin’s lymphoma cell lines with different sensitivity to cytotoxic drugs. Exp Hematol (2008) 36:886–96. 10.1016/j.exphem.2008.02.014 18400362

[44] Hoennscheidt C Max D Richter N Staege MS. Expression of CD4 on Epstein-Barr virus-immortalized B cells. Scand J Immunol (2009) 70:216–25. 10.1111/j.1365-3083.2009.02286.x 19703011

[45] Foell JL Volkmer I Giersberg C Kornhuber M Horneff G Staege MS. Loss of detectability of Charcot-Leyden crystal protein transcripts in blood cells after treatment with dimethyl sulfoxide. J Immunol Methods (2008) 339:99–103. 10.1016/j.jim.2008.08.006 18789335

[46] Hesse M Willscher E Schmiedel BJ Posch S Golbik RP Staege MS. Sequence and expression of the chicken membrane-associated phospholipases A1 alpha (LIPH) and beta (LIPI). Mol Biol Rep (2012) 39:761–9. 10.1007/s11033-011-0796-0 21559832

[47] Kovar H Dworzak M Strehl S Schnell E Ambros IM Ambros PF Overexpression of the pseudoautosomal gene MIC2 in Ewing’s sarcoma and peripheral primitive neuroectodermal tumor. Oncogene (1990) 5:1067–70. 1695726

[48] Matsumoto Y Tanaka K Nakatani F Matsunobu T Matsuda S Iwamoto Y. Downregulation and forced expression of EWS-Fli1 fusion gene results in changes in the expression of G(1)regulatory genes. Br J Cancer (2001) 84:768–75. 10.1054/bjoc.2000.1652 11259090 PMC2363806

[49] Staege MS Hattenhorst UE Neumann UE Hutter C Foja S Burdach S. DNA-microarrays as tools for the identification of tumor specific gene expression profiles: applications in tumor biology, diagnosis and therapy. Klin Padiatr (2003) 215:135–9. 10.1055/s-2003-39371 12838936

[50] Boon T Cerottini JC Van den Eynde B van der Bruggen P Van Pel A. Tumor antigens recognized by T lymphocytes. Annu Rev Immunol (1994) 12:337–65. 10.1146/annurev.iy.12.040194.002005 8011285

[51] Butt AQ Mills KH. Immunosuppressive networks and checkpoints controlling antitumor immunity and their blockade in the development of cancer immunotherapeutics and vaccines. Oncogene (2014) 33:4623–31. 10.1038/onc.2013.432 24141774

[52] Crespo J Sun H Welling TH Tian Z Zou WT Cell anergy, exhaustion, senescence, and stemness in the tumor microenvironment. Curr Opin Immunol (2013) 25:214–21. 10.1016/j.coi.2012.12.003 23298609 PMC3636159

[53] Harding FA McArthur JG Gross JA Raulet DH Allison JP CD28-mediated signaling costimulates murine T cells and prevents induction of anergy in T-cell clones. Nature (1992) 356:607–9. 10.1038/356607a0 1313950

[54] Davis ID. An overview of cancer immunotherapy. Immunol Cell Biol (2000) 78:179–95. 10.1046/j.1440-1711.2000.00906.x 10849106

[55] Nanda NK Sercarz EE Induction of anti-self-immunity to cure cancer. Cell (1995) 82:13–7. 10.1016/0092-8674(95)90047-0 7606778

[56] Uyttenhove C Maryanski J Boon T. Escape of mouse mastocytoma P815 after nearly complete rejection is due to antigen-loss variants rather than immunosuppression. J Exp Med (1983) 157:1040–52. 10.1084/jem.157.3.1040 6187879 PMC2186952

[57] Garrido F Ruiz-Cabello F Cabrera T Perez-Villar JJ Lopez-Botet M Duggan-Keen M Implications for immunosurveillance of altered HLA class I phenotypes in human tumours. Immunol Today (1997) 18:89–95. 10.1016/S0167-5699(96)10075-X 9057360

[58] Ochoa AC Longo DL. Alteration of signal transduction in T cells from cancer patients. Important Adv Oncol (1995) 1995:43–54. 7672813

[59] Lafferty KJ Gill RG. The maintenance of self-tolerance. Immunol Cell Biol (1993) 71:209–14. 10.1038/icb.1993.23 8349303

[60] Kühnöl C Herbarth M Föll J Staege MS Kramm C. CD137 stimulation and p38 MAPK inhibition improve reactivity in an in vitro model of glioblastoma immunotherapy. Cancer Immunol Immunother (2013) 62:1797–809. 10.1007/s00262-013-1484-9 24129764 PMC11028552

[61] Linch SN McNamara MJ Redmond LW. OX40 agonists and combination immunotherapy: putting the pedal to the metal. Front Oncol (2015) 5:34. 10.3389/fonc.2015.00034 25763356 PMC4329814

[62] Andarini S Kikuchi T Nukiwa M Pradono P Suzuki T Ohkouchi S Adenovirus vector-mediated in vivo gene transfer of OX40 ligand to tumor cells enhances antitumor immunity of tumor-bearing hosts. Cancer Res (2004) 64:3281–7. 10.1158/0008-5472.CAN-03-3911 15126371

[63] Weinberg AD OX40: targeted immunotherapy – implications for tempering autoimmunity and enhancing vaccines. Trends Immunol (2002) 23:102–9. 10.1016/S1471-4906(01)02127-5 11929124

[64] Ali SA Ahmad M Lynam J McLean CS Entwisle C Loudon P Anti-tumour therapeutic efficacy of OX40L in murine tumour model. Vaccine (2004) 22:3585–94. 10.1016/j.vaccine.2004.03.041 15315837

[65] Piconese S Valzasina B Colombo MP. OX40 triggering blocks suppression by regulatory T cells and facilitates tumor rejection. J Exp Med (2008) 204:825–39. 10.1084/jem.20071341 18362171 PMC2292222

[66] Cuadros C Dominguez AL Lollini PL Croft M Mittler RS Borgstrom P Vaccination with dendritic cells pulsed with apoptotic tumors in combination with anti-OX40 and anti-4-1BB monoclonal antibodies induces T cell-mediated protective immunity in Her-2/neu transgenic mice. Int J Cancer (2005) 116:934–43. 10.1002/ijc.21098 15856473

[67] Pan PY Zang Y Weber K Meseck ML Chen SH. OX40 ligation enhances primary and memory cytotoxic T lymphocyte responses in an immunotherapy for hepatic colon metastases. Mol Ther (2002) 6:528–36. 10.1006/mthe.2002.0699 12377195

[68] Verhoeven DH de Hooge AS Mooiman EC Santos SJ ten Dam MM Gelderblom H NK cells recognize and lyse Ewing sarcoma cells through NKG2D and DNAM-1 receptor dependent pathways. Mol Immunol (2008) 45:3917–25. 10.1016/j.molimm.2008.06.016 18657862

[69] Cho D Shook DR Shimasaki N Chang YH Fujisaki H Campana D. Cytotoxicity of activated natural killer cells against pediatric solid tumors. Clin Cancer Res (2010) 16:3901–9. 10.1158/1078-0432.CCR-10-0735 20542985 PMC3168562

